# Is fascia lata a viable graft for MPFL reconstruction? An overview of surgical technique and scoping literature review

**DOI:** 10.1097/MS9.0000000000002731

**Published:** 2024-11-14

**Authors:** Dimitrios Zachariou, Panagiotis Karampinas, Iordanis Varsamos, Athanasios Galanis, Michail Vavourakis, Ioannis Spyrou, Evangelos Sakellariou, Christos Patilas, Georgios Tsalimas, Angelos Kaspiris, John Vlamis, Spiros Pneumaticos

**Affiliations:** Third Department of Orthopedic Surgery, National and Kapodistrian, University of Athens, KAT General Hospital, Athens, Greece

**Keywords:** fascia lata graft, lateral patellar instability, medial patellofemoral ligament, MPFL reconstruction, patellar dislocation

## Abstract

Lateral patellar dislocation is irrefutably one of the most common knee injuries, while subsequent medial patellofemoral ligament (MPFL) damage requires proper orthopedic care. Treatment of these injuries is regularly associated with the need for MPFL reconstruction surgery. This operation, often in combination with other procedures, can result very fruitful in restoring knee kinematics. There is a considerable variety of options for the orthopedic surgeon to choose from regarding graft selection when performing MPFL reconstruction surgery, including the semitendinosus tendon, the gracilis, and others. Notwithstanding, utilizing the fascia lata as an autograft or allograft for MPFL reconstruction has emerged as a promising alternative. Perusing the existing literature, not any review paper could be found examining the efficacy of fascia lata as a MPFL reconstruction graft. As a result, a thorough search was conducted in various databases to investigate and explore the studies analyzing this type of MPFL reconstruction surgery. The number of papers scrutinizing this operation was exceedingly narrow. However, out of these studies, it can be concluded that opting for fascia lata grafts when executing MPFL reconstruction surgery features some considerable advantages, involving similar biomechanics to the native MPFL, no hamstrings damage and quicker rehabilitation among others. This paper accentuates the requirement for considering fascia lata as a viable graft option in MPFL reconstruction surgery and the necessity for more pertinent research in order to attain more reliable inferences.

## Introduction

Highlights

- Lateral patellar dislocation is irrefutably one of the most common knee injuries.
- Treatment of these injuries is regularly associated with the need for MPFL reconstruction surgery.
- There is a considerable variety of options for the orthopedic surgeon to choose from regarding graft selection when performing MPFL reconstruction surgery, including the semitendinosus tendon, the gracilis, and others.
- Utilizing the fascia lata as an autograft or allograft for MPFL reconstruction has emerged as a promising alternative.
- In the existing literature, not any review paper could be found examining the efficacy of fascia lata as a MPFL reconstruction graft.
- Indubitably, fascia lata features some substantial advantages over the other grafts, such as similar biomechanics to the native MPFL, leaving the hamstrings intact, and, in the case of allograft use, no donor site morbidity, swifter rehabilitation and reduced operative time.
- Although results appear to be highly promising, further apposite research is required to accomplish more solid deductions.

Patellar instability is a common knee disorder that occurs between the ages of 10–20 years, with up to 23 patellofemoral dislocations per 100 000 people approximately^1^. Its occurrence depends on various factors, including female sex, young age, patella alta, lateral patellar tilt, and trochlear dysplasia, Q angle and genu valgum, vastus medialis muscle hypoplasia, ligament hyperlaxity, external tibial torsion, subtalar pronation, or excessive femoral anteversion. All these factors can contribute to the high prevalence of patellar dislocations^2^.

The stability of the patellofemoral joint is determined by both bony and soft tissue constraints. The medial patellofemoral ligament (MPFL) is an important restraint against lateral subluxation or dislocation of the patella. The MPFL and other soft tissue restraints, involving the medial patellomeniscal ligament, medial patellotibial ligament, medial retinaculum, and even lateral retinaculum, are all vitally important contributors to patellofemoral stability^3^. Nonetheless, it is the MPFL that is usually most clearly damaged, whilst it has been proven to fail in more than 90% of cases following acute lateral patellar dislocations^1^.

Diagnosing patellar instability can be a complex process that necessitates a rigorous clinical examination. A dislocated patella must first be reduced by fully extending the knee, followed by ‘pushing’ the patella medially^1–3^. Furthermore, a proper physical examination should assess the range of motion, apprehension sign, and patellar compression pain. A positive sign of patellar instability is indicated when the patella, divided into four areas longitudinally on the coronal plane, features lateralization of more than 2/4^4^. Nevertheless, it is essential to conduct a MRI examination to scrutinize the MPFL and the other medial knee stabilizers to corroborate the diagnosis. A computerized tomography (CT) investigation must be carried out in cases of high suspicion of underlying knee conditions coexisting with the injury, such as trochlear dysplasia or tibial tubercle malalignment^5,6^.

The standard treatment approach for most first-occurred patellar dislocations involves nonoperative management with resting and rehabilitation, however, these injuries can trigger articular cartilage damage, osteochondral fractures, and recurrent instability^7,8^. Surgical treatment commonly includes MPFL reconstruction, occasionally in conjunction with other apposite procedures such as lateral retinacular release, tibial tubercle osteotomy, or trochleoplasty^9^.

Pertinent graft selection is of paramount significance in the reconstruction of knee ligaments, such as the anterior (ACL) or posterior (PCL) cruciate ligament^10,11^. MPFL reconstruction techniques include tendon transfers, autografts, and synthetic grafts^12–14^. While the gracilis tendon is customarily utilized, its tubular shape differs from the native MPFL shape. Consequently, it appears to be more robust and stiffer compared to the native ligament, which is more sheet-like^10–14^. Notwithstanding, the fascia lata, which can be used as both an allograft and an autograft for MPFL reconstruction, has been denoted to feature promising outcomes as an excellent alternative to the gracilis tendon due to its properties and shape, offering a potentially brighter future for MPFL reconstruction^15,16^.

This paper aims to provide a comprehensive understanding of the present-day evidence on techniques and outcomes following MPFL reconstruction employing a fascia lata autograft or allograft, while bolstering orthopedic surgeons’ knowledge surrounding this surgical approach. To our knowledge, this is the first review scrutinizing the surgical option of MPFL reconstruction with fascia lata grafts.

## Materials and methods

**Figure 1:**
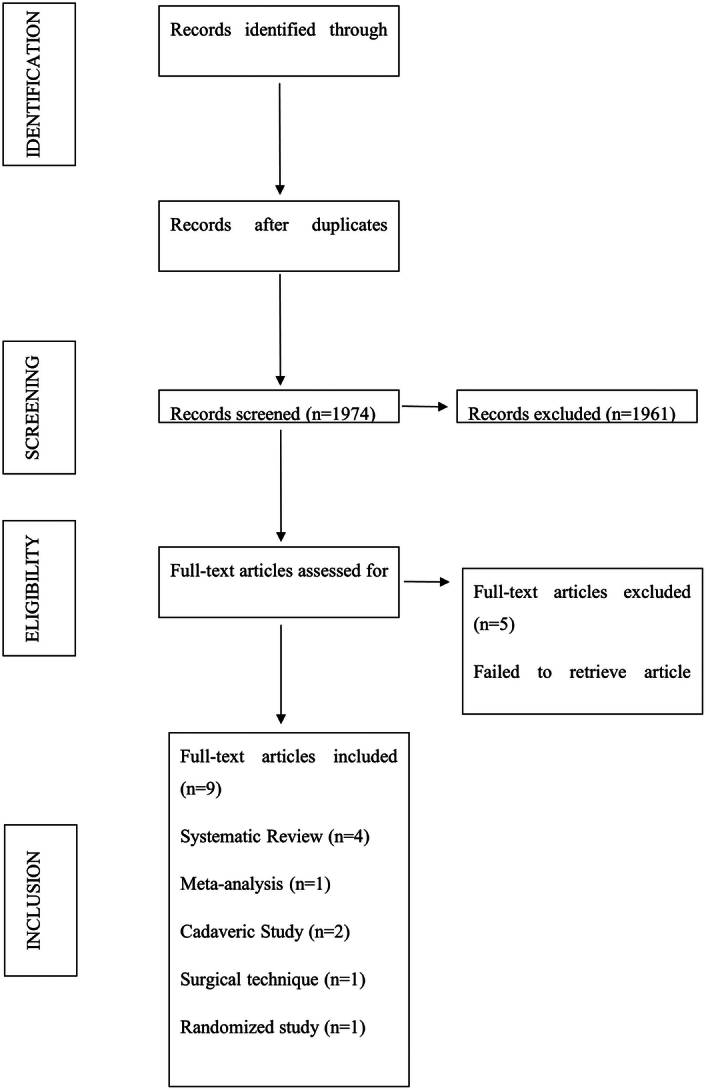
Study’s flowchart.

A diligent literature research was executed employing the MEDLINE/PubMed, Google Scholar, Web of Science, and Embase databases for articles published between 2000 and 2024. Keyword search terms utilized were: ‘MPFL’, ‘Medial patellofemoral ligament’, ‘lateral patellar instability’, ‘reconstruction’, ‘patellar dislocation’, ‘fascia lata’, and ‘graft’. Language filters were activated for English. No restrictions were imposed concerning the scientific articles’ publication date. In terms of inclusion criteria, they were clinical studies, case series, reviews, and papers reporting data apposite to our topic. Contrariwise, exclusion criteria were papers about MPFL reconstruction that did mention fascia lata autografts or allografts. Articles in full text were explored to retrieve additional relevant papers (Fig. 1).

### MPFL specifics and fascia lata reconstruction surgery

#### MPFL anatomy

Back in 1957, Kaplan identified fibers that matched the description of the MPFL but did not name them as such. Warren and Marshall later named these ligaments in 1979^17^. Copious anatomical studies characterize the MPFL as featuring a smaller femoral origin and a broad, fan-shaped insertion on the patella and quadriceps. Nomura *et al*.^5^ delineated two parts of the MPFL: an inferior straight and a superior oblique, the latter closely linked to the Vastus Medialis Oblique. The origin of the MPFL varies across studies. Still, it is commonly located near the adductor tubercle and medial epicondyle, typically in the ‘saddle’ area between these points or within 1 cm distal to the adductor tubercle^18^. The superior fibers blend with the patellar tendon and the Vastus Medialis Oblique (VMO)^5,19^. Owing to anatomical variability, the femoral origin of the MPFL is frequently considered a ‘cloud’ rather than a specific ‘point’^20^.

#### MPFL biomechanics

The MPFL is broadly responsible for 50–80% of the resistance against lateral patellar glide^21^. When a lateral patellar dislocation does take place, the MPFL becomes ineffective, enhancing the likelihood of future dislocations. Other structures, such as the patellomeniscal and patellotibial ligaments and the superficial medial retinaculum, contribute less significantly to patellar stability^4^. The VMO muscle plays a crucial role as a dynamic stabilizer. The patella’s movement and stability in the patellofemoral joint are maintained through the synergetic effect between these static and dynamic stabilizers^20,21^. The patella’s path within the femoral trochlea involves tilting, gliding, and rotating. In full knee extension, the patella rests above the femoral sulcus and moves into the trochlea at 10–30° of knee flexion, which depends on the length of the patellar tendon. This entry is delayed in cases of patella alta or a ‘short’ trochlea, resulting in inadequate osteoarticular stabilization when the knee is extended or slightly flexed^8,22^.

#### MPFL grafts

Graft selection is ordinarily regarded as a critical decision regarding knee ligament reconstruction. However, graft choice in MPFL reconstruction has received less focus in the existing literature^2,4,17,21^. Standard graft options include semitendinosus, gracilis, tibialis anterior and posterior, peroneus, quadriceps, patellar, and adductor tendons. Both autografts and allografts have been used in MPFL reconstruction, with the gracilis tendon being the most commonly utilized autograft and the semitendinosus-gracilis the most frequently employed allograft^23^. Newly, the fascia lata, introduced as both an allograft and autograft for MPFL reconstruction, has emerged as a considerably promising alternative to the gracilis tendon^24,25^.

While the gracilis tendon is frequently used, its tubular shape differs from the native MPFL^26^. Hence, it is sturdier and stiffer than the native sheet-like ligament, which resembles the fascia lata graft^15,16^. Besides that, the fascia lata also enables orthopedic surgeons to customize the graft size in accordance with the patient’s requirements and offers the option to preserve the hamstrings, therefore securing a secondary medial stabilizer. Finally, it can be used as an alternative graft in revision cases where the hamstrings have already been harvested^16^.

#### Surgical technique

A 6 cm incision is initially made on the lateral thigh, ~5 cm above the upper border of the patella. After blunt dissection down to the fascia lata, a section of roughly 10 cm in length and 1 cm in width is marked. This marked portion of the fascia is gingerly detached from the surrounding soft tissues, while maintaining tension on the graft to avoid tearing. Once freed, the remaining gap in the fascia is suture-closed. The harvested autograft is then carefully prepared.

**Figure 2:**
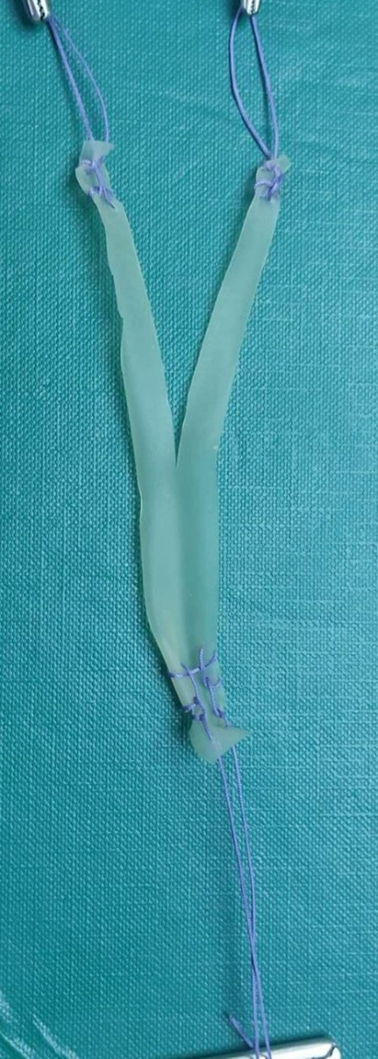
Visualization of the final graft form using a Penrose drain.

The graft is split into two sections with a scalpel up to the midpoint, creating a Y-shaped graft. It is advisable to suture the middle of the graft to prevent further autograft splitting (Fig. 2).

Since the patellar MPFL insertion extends from the superomedial corner of the patella to the midpoint of the medial patellar border, two holes need to be drilled in the proximal two-thirds of the medial side^27^. Extensive attentiveness is requisite to avoid fracturing the patella and/or injuring the chondral surface. After drilling the two 4 mm width and 25 mm deep tunnels, each leg of the graft’s double-strand end is inserted into the respective hole and secured with a knotless anchor.

The femoral MPFL insertion is slightly anterior to the extension of the posterior femoral cortex, between the medial condyle’s proximal origin and the most posterior point of Blumensaat’s line^28^. A drill hole, 0.5 mm wider than the graft ends, is created up to the contralateral cortex. The graft is then pulled into the femoral tunnel using a second suture loop and fixed with a biodegradable interference screw, matching the tunnel’s diameter, at 30° of knee flexion.

Several studies have indicated that the MPFL provides maximum restraint against lateral patella displacement at 30° of knee flexion^29^. The full range of motion and patellar tracking are checked between 0° and 40° of knee flexion. Proper restoration of medial restraint should prevent lateral patellar dislocation, but overcorrection should be avoided to avert medial subluxation, graft failure, or medial patellofemoral pressure syndrome.

## Discussion

Multiple grafts have been propounded and utilized for the reconstruction of the MPFL. Lately, fascia lata grafts have been successfully used both as autografts and allografts for anatomic reconstruction of the MPFL, demonstrating their effectiveness^24,25^. The fascia lata features multiple advantages over other types of grafts used for MPFL reconstruction.

To begin with, it has a comparable shape and biomechanical similarity to the native MPFL. Likewise, it resembles the shape of an aponeurosis, contrary to the tubular shape of the gracilis or semitendinosus graft, which are also more robust and stiffer than the native MPFL, thus being unable to replace the MPFL functional behavior^25,26^.

In 2016, a study by Stephen *et al*. examined the gracilis, the quadriceps, and the tensor fasciae lata grafts for MPFL reconstruction in cadavers. These three grafts have been suggested to exhibit the closest strength and stiffness to the native MPFL. In all cases, after achieving an anatomic placement of the femoral tunnel and the graft tensioned to 2 N, they observed similar lateral patellar tilt, lateral patellar translation, peak and mean joint contact pressures and peak and mean lateral joint pressures to the native MPFL^30^.

On the other hand, Lorbach *et al*., in their cadaveric study, contrasted the patellofemoral contact pressure with native MPFL and the reconstructed one utilizing a pressure film. The reconstruction was performed with either a gracilis tendon or a fascia lata graft. Yet, the contact pressure was not fully restored to that of the native MPFL in either group with the anatomical placement of the bone tunnels. This interesting study also revealed a dynamic patellofemoral contact pressure reduction in both groups. In their juxtaposition, they did not report a considerable superiority of one graft over the other, connoting that the fascia lata graft can replace gracilis grafts in MPFL reconstruction^16^.

Another study by Matuszewski *et al*. in 2017 examined the use of fascia lata allograft and gracilis tendon autograft in the reconstruction of the MPFL in children. They reported no noteworthy healing problems or graft insufficiency, except for one case of mild superficial infection in the fascia lata group. Also, a single recurrence was mentioned but was attributed to anchor loosening, not graft failure. Even though the effectiveness of the grafts was similar, the median duration of the operation was lower by 20 min in the fascia lata group. Additionally, employing cadaveric fascia lata allograft allowed for sparing of the hamstring muscles, which act as dynamic stabilizers of the medial knee or possible grafts in other surgical procedures, thus allowing for quicker rehabilitation and less donor site morbidity. Matuszewski *et al*.^31^, similarly to the other cadaveric studies, drew the inference that using fascia lata allograft can yield safe and reliable results.

What is more, Muccioli *et al*. 2021 performed revision MPFL reconstruction surgery in nineteen patients utilizing fascia lata allografts. Seventeen of them were available at the 60-month follow-up. They demonstrated significant improvement postoperatively with restored patellar stability, 94% improvement in knee function and no cartilage deterioration. No notable amelioration was discerned between the 24 and 60-month follow-up. The reported failure rate was just 6%, owing to a recurrence at 25 months postoperative that required revision surgery^32^ (Table 1).

**Table 1:** Cross-table between existing studies.

Author and year	Type of study (*N*)	Type of graft (*N*)	Follow-up (months)	Outcome
Stephen *et al*. (2016)^30^	Cadaveric study (9)	Gracilis tendon (9), Quadriceps tendon (9), fascia lata (9)	N/A	Similar lateral patellar tilt, lateral patellar translation, peak and mean joint contact pressures and peak and mean lateral joint pressures to the native MPFL
Lorbach *et al*. (2017)^16^	Cadaveric study (8)	Gracilis tendon (8), fascia lata (8)	N/A	The contact pressure was not fully restored compared to native MPFL in either group. No significant superiority of one graft over the other
Matuszewski *et al*. (2017)^31^	Randomized study (44)	Gracilis tendon (autograft) (22), fascia lata (allograft) (22)	24	Similar effectiveness of the grafts, lower median duration with fascia lata, a case of mild superficial infection in the fascia lata group, single recurrence due to anchor loosening
Muccioli *et al*. (2021)^32^	Case series (19)	Fascia lata (allograft) (19)	60	Significant improvement postoperative, 94% improvement in knee function, no cartilage deterioration

ACL, anterior cruciate ligament; CT, computed tomography; MPFL, medial patellofemoral ligament; PCL, posterior cruciate ligament; VMO, vastus medialis oblique.

Another vitally important aspect is that fascia lata autograft can also be considered a viable option in revision cases where the gracilis tendon has already been harvested. In addition, it enables the operating surgeon to harvest a graft corresponding to the native MPFL and in accordance with the anatomic requirements by adjusting the graft’s width and length^16^.

It should be underlined that special care should be taken in terms of the placement of the femoral tunnel. Previous studies have revealed that a nonanatomical positioning can pronouncedly alter the contact pressure^30^. A paper by Schottle *et al*.^28^ in 2007 described the tunnel’s optimal positioning, which allowed for optimal outcomes following MPFL reconstruction surgery. Still, even with the so-called anatomic MPFL reconstruction, it might not fully reinstate the native knee kinematics^16^.

## Conclusion

There is a plethora of grafts to select from when performing MPFL reconstruction surgery. Indubitably, fascia lata features some substantial advantages over the other grafts, such as similar biomechanics to the native MPFL, leaving the hamstrings intact, and, in the case of allograft use, no donor site morbidity, swifter rehabilitation and reduced operative time. Still, much care should be taken regarding the placement of the tunnels since it has been proven to exert an influence on the results of the MPFL reconstruction surgery significantly. The number of papers that scrutinize the utilization of fascia lata in MPFL reconstruction is very limited in the existing literature. Although results appear to be highly promising, further apposite research is required to accomplish more solid deductions.

## Ethical approval

Ethics approval was not required for this review.

## Consent

Informed consent was not required for this review.

## Source of funding

No funding was received to conduct this study.

## Author contribution

D.Z.: conceptualization and writing – original draft; P.K.: visualization and writing – original draft; I.V. and A.G.: methodology and data curation; M.V. and I.S.: formal analysis and investigation; E.S.: resources; C.P.: software; G.T.: validation; A.K.: project administration and writing – review and editing; J.V.: conceptualization and writing – review and editing; S.P.: supervision.

## Conflicts of interest disclosure

The authors declare no conflicts of interest.

## Research registration unique identifying number (UIN)

Name of the registry: not applicable.
Unique identifying number or registration ID: not applicable.
Hyperlink to your specific registration (must be publicly accessible and will be checked): not applicable.

## Guarantor

Dimitrios Zachariou.

## Data availability statement

All raw data are available to access should they be requested.

## Provenance and peer review

Paper was not invited.
